# Effect of pulsed light on curcumin chemical stability and antioxidant capacity

**DOI:** 10.1371/journal.pone.0291000

**Published:** 2023-09-01

**Authors:** Huiying Amelie Zhang, Anubhav Pratap-Singh, David D. Kitts

**Affiliations:** Faculty of Land of Food Systems, Food Science, Food, Nutrition and Health, University of British Columbia, Vancouver, BC, Canada; University of Brescia: Universita degli Studi di Brescia, ITALY

## Abstract

Curcumin is the major bioactive component in turmeric with potent antioxidant activity. Little is known about how pulsed light (PL) technology (an emerging non-thermal food processing technology relying on high intensity short duration flashes of light) can affect the chemical stability and antioxidant capacity of curcumin. This study found that PL treatment of fluence levels from 0 to 12.75 J/cm^2^ produced a fluence-dependent reduction in curcumin content. These results paralleled the production of a tentative curcumin dimer, identified as a potential photochemical transformation product. PL-treated curcumin at relatively higher fluence levels decreased chemical-based ORAC and ABTS antioxidant capacity, relative to control (*P* < 0.05). This contrasted the effect observed to increase coincidently both intracellular antioxidant capacity (e.g., DCFH-DA (*P* < 0.05)) and GSH/GSSG ratio (*P* < 0.05), respectively, in cultured differentiated Caco-2 cells. In conclusion, the application of PL on curcumin results in photochemical transformation reactions, such as dimerization, which in turn, can enhance biological antioxidant capacity in differentiated Caco-2 cells.

## Introduction

Curcuminoids are a major group of bioactive polyphenolic compounds present in turmeric, and among the curcuminoids, curcumin is the principal component (77% of total curcuminoids) and the one with the most potent antioxidant capacity reported in both selective chemical and cell systems [1–4]. These facts have led to the important question concerning the chemical stability of curcumin when exposed to different processing and storage conditions. Previous studies have shown that curcumin is sensitive to alkaline pH [5], high temperatures > 160°C [6], autooxidation and exposure to reactive oxygen species [7,8], and light exposure [9,10].

The PL technology stands out from traditional continuous-wave light technologies for its use of light pulses of high intensity (e.g., up to 1000-fold instantaneous energy compared to continuous wave UV light) and short duration (e.g., in microseconds) to achieve promising food processing applications in a short treatment time (e.g., in seconds or minutes) [11–13]. The effects of PL on phenolic compounds that are commonly present in vegetables and fruits have been studied by many research groups, but the findings are not completely consentaneous. Many studies reported PL application resulted in an increase in total phenolics content (TPC) in vegetables and fruits [14–18], while the lack of significant changes [19,20] or decrease in TPC [21,22] have also been reported. The total fluence (which describes the total radiant energy that is received from the light source by the matrix per unit area during the entire treatment time) is regarded as an important factor governing TPC stability, as evidenced in PL treatment model Gallic acid solution were both TPC and free radical scavenging activity were reduced at high fluence application [23].

The photosensitivity of curcumin has been studied under sunlight, visible light, and ultraviolet (UV) light. Both hydrolytic (breakdown of the heptane chain that connects the two aromatic rings due to photodegradation) and oxidative (photosensitization of molecular oxygen and production of reactive oxygen species and curcumin radicals due to photooxidation) reactions were reported as photochemical transformation mechanisms of curcumin [24–26]. The composition and relative abundance of consequent products differ depending on the physical state of curcumin and the conditions of the light source [24]. Vanillin, ferulic aldehyde, ferulic acid, vanillic acid, and 4-vinylguaiacol are photodegradation products of curcumin [27]. Visible light irradiation on solubilized curcumin generates reactive oxygen species (ROS) such as superoxide anion radical (O_2_^•–^) and singlet molecular oxygen (^1^O_2_), via electron and energy transfer, respectively [26]. In addition, curcumin radicals were identified in this process [28]. As the initiation of curcumin oxidation involves ROS attack and formation of curcumin radicals [7], we hypothesize that photooxidation of curcumin induced by photo-generated ROS may produce similar products as those reported for the oxidative transformations of curcumin. Examples of such products include cyclization products (bicyclopentadione, diguiacol, cyclobutylcyclopentadione, dihydrocyclopentadione, ketohydroxycyclopentadione, and hemiacetalcyclopentadione) and dimerization products (curcumin dimers) [7,8,29,30]. So far, the photochemical stability, degradation kinetics and products of curcumin have been studied only under sunlight, visible, and UV light conditions. With pulsed light emerging as an attractive non-thermal light-based food processing technology, more information on its effect on the photostability of curcumin is required.

This study aimed to quantify the effect of PL treatment applied at levels typical of a commercial operation, on curcumin content in a model curcumin solution, and to identify the relationship between the changes in curcumin content with changes in chemical and biological antioxidant capacity. Identification of possible photochemical transformation products was also performed to better understand the changes observed in the chemical and biological measures of antioxidant capacity related to curcumin transformation.

## Materials and methods

### Materials

Methanol for sample preparation and HPLC-grade solvents were purchased from VWR International (Edmonton, AB, Canada). Pure curcumin standard (Product No. 78246, ≥99.5%), was purchased from Sigma-Aldrich (St. Louis, MO, USA). Deuterium-labelled curcumin standard, namely d_6_-curcumin, was purchased from C/D/N Isotopes (Pointe-Claire, QC, Canada). Most chemicals and reagents that were required for chemical- and cell-based antioxidant assays and cell viability assays were purchased from Sigma-Aldrich, while the glutathione Assay Kit was purchased from Cayman Chemical (Item No. 703002; Ann Arbor, MI, USA). Caco-2 cells used in this study, were obtained from American Type Culture Collection (ATCC; Manassas, VA, USA). Cell culture dishes and plates were purchased from Sarstedt AG & Co. KG (Nümbrecht, Germany). Dulbecco’s modified Eagle’s medium (DMEM; Product No. D5796), Dulbecco’s Phosphate Buffered Saline (PBS; Product No. D8537), Penicillin-Streptomycin (Product No. P0781) came from Sigma-Aldrich. Fetal Bovine Serum (FBS; Product No. 10437028) and Trypsin-EDTA (0.05%), phenol red (Product No. 25300062) produced by Gibco™ were purchased from Fisher Scientific.

### Sample preparation and PL treatment

For PL treatment, 1 mL of 1 mM methanolic solution of curcumin was contained in a 2.8-cm diameter clear glass and covered with a clear glass lid. The sample was placed under the center of a PL lamp, 5 cm from the light source. Fluence per pulse was 0.017 J/cm^2^, as measured by a radiometer. A central composite design was used for PL settings (**Table 1**)–frequency was varied from 1–7 Hz and treatment time was varied from 2–146 s, resulting in a total of 10 fluence levels (0.78–12.75 J/cm^2^) with two center points (5.03 J/cm^2^)–all variables and combinations were relevant to a food processing protocol, with extreme conditions also tested to a relatively lesser amount. For all analyses, curcumin without PL treatment was included as a control. Fluence was calculated using Eq (1):

$$Total\;fluence\;(J/{cm}^{2})=Fluence\;per\;pulse\;(J/{cm}^{2})\times Frequency\;(Hz)\times Time\;(s)$$ (1)

**Table 1:** Pulsed light settings in Central Composite Circumscribed (CCC) design.

Standard run order	Factor A (Frequency)		Factor B (Time)		Fluence (J/cm^2^)
	Coded value	Actual Value (Hz)	Coded Value	Actual Value (s)	
1	-1	2	-1	23	0.78
2	-1	2	1	125	4.25
3	1	6	-1	23	2.35
4	1	6	1	125	12.75
5	-1.414	1	0	74	1.26
6	1.414	7	0	74	8.81
7	0	4	-1.41	2	0.14
8	0	4	1.41	146	9.93
9 (C)	0	4	0	74	5.03
10 (C)	0	4	0	74	5.03

(C) indicates the center points of the Central Composite Design.

### Quantification of curcumin content by HPLC-DAD

An Agilent 1100 high-performance liquid chromatography (HPLC) system was used to quantify the content of curcumin in untreated and PL-treated samples. The system was equipped with an Agilent ZORBAX SB-C18 column (150 × 4.6 mm i.d., 5 μm particle size; Santa Clara, CA, USA) as the stationary phase. The mobile phase consisted of a mixture of water and acetonitrile (55:45 v/v) with 0.1% (v/v) formic acid, and the flow rate was 1.0 mL/min. Sample injection volume was 20 μL. The compounds were detected with a diode-array detector (DAD) at 425 nm after isocratic elution using the mobile phase. According to preliminary experiments using these specific conditions, curcumin has a retention time of ~11.5 min. A standard curve of CUR (Eq 2, R^2^ = 0.9998) was developed for the quantification of the curcumin content in control and samples:

$$Curcumin\;content\;(\mu M)=45.087\times Peak\;Area\;({mAU}^{*}s)$$ (2)

### Identification of potential curcumin photo-transformation products

The determination of curcumin and potential photo-transformation products (**S1 Table** in S1 File) was performed using an Agilent 1200 HPLC system equipped with G4212A diode-array detection (DAD) and 6530B quadrupole time-of-flight (QTOF) detectors (Agilent, Santa Clara, CA, USA). The HPLC conditions for the separation of the phenolic compounds were performed according to Pico et al. [31] with slight modifications. Briefly, the separation was accomplished using a Zorbax SB-C18 column (50 mm length x 4.6 mm internal diameter, 5 μm particle size; Agilent) equipped with a Zorbax SB-C18 guard column (12.5 mm length x 4.6 mm internal diameter, 5 μm particle size; Agilent), both thermostated at 40°C with a constant flow rate of 0.8 mL/min and a gradient of water with 2% formic acid (v/v) (solvent A) and acetonitrile with 0.1% formic acid (v/v) (solvent B). The gradient of mobile phase is presented in **S2 Table** in S1 File. The injection volume was 5 μL, and the tray was thermostated at 4°C to avoid degradation of phenolic compounds. D_6_-curcumin and d_3_-vanillin (10 μg/mL in methanol) were used as internal standards of curcumin and potential photo-transformation products, respectively.

The MS mode conditions of the QTOF detector were as follow: ionization mode in ESI (+); drying gas (nitrogen) flow and temperature of 5 mL. min^-1^ and 325°C, respectively; sheath gas (nitrogen) flow and temperature of 12 mL. min^-1^ and 275°C, respectively; nebulizer pressure of 40 psi; capillary and nozzle voltages of 2,500 and 2,000 V, respectively; fragmentor, skimmer, and octapole voltages of + 200, 65, and 750 V, respectively.

To identify other potential photo-transformed curcumin products for which analytical standards were not available (**S3 Table** in S1 File), the QTOF detector was also operated in Product Ion mode (*i*.*e*., MS/MS). Following the same HPLC and QTOF conditions as for the MS mode, the collision gas (nitrogen) cell was activated and defined at 20 psi and 35 eV. As there was only one compound that was not present in the untreated curcumin control but was found in all except one of the PL-treated samples (peak with m/z 735.2436 at retention time 18.479 min), this compound and curcumin were the only ions included in the MS/MS method with the collision cell conditions above-mentioned.

### ABTS assay

The ABTS assay was performed according to the protocol reported previously [32] with minor modifications. ABTS radical cations (ABTS^•+^) was generated by reacting ABTS (7 mM) with potassium persulfate (2.45 mM) at a 1:1 (v/v) ratio for at least 12 h at room temperature in the dark. The ABTS^•+^ solution was then diluted with PBS (pH 7.4) to obtain an absorbance of 0.70 ± 0.02 at 734 nm, and the diluted solution was referred to as ABTS^•+^ working solution hereafter. ABTS^•+^ working solution was prepared fresh prior to each assay. 90 μL/well of ABTS^•+^ working solution was added to 96-well microplates and absorbance at 734 nm was measured, followed by addition of 10 μL of Trolox (a vitamin E analogue that served as the antioxidant standard; 0–200 μM), or curcumin samples (untreated control or PL-treated ones; 25–100 μM). After 6 min, absorbance at 734 nm was measured using a Thermo Labsystems Multiskan Spectrum microplate spectrophotometer (Thermo Fisher Scientific, Waltham, MA, USA). The radical scavenging capacity of sample or Trolox is calculated using Eq (3), and the antioxidant capacity of sample is expressed as μmol Trolox Equivalent (TE)/μmol sample, calculated as the ratio between the slope of %ABTS^•+^ Scavenging linear regression equation for the curcumin sample to that of Trolox antioxidant standard:

$${\%\;ABTS}^{•+}\;Scavenging=\frac{{Abs}_{734\;nm}(Control)-{Abs}_{734\;nm}(Sample\;OR\;Trolox)}{{Abs}_{734\;nm}(Control)}$$ (3)

where Abs_734nm_(Control) is the absorbance at 734 nm of ABTS^•+^ working solution prior to addition of sample or Trolox, Abs_734nm_(Sample OR Trolox) is the absorbance at 734 nm at 6 min after addition of sample or Trolox, respectively.

### ORAC assay

The total antioxidant capacity of samples was assessed using the oxygen radical absorbance capacity fluorescein (ORAC) assay using the protocol developed previously [33]. A 75 mM phosphate buffer (PB) was prepared by mixing 0.75 M K_2_HPO_4_ and 0.75 M NaH_2_HPO_4_ in the ratio of 61.6:38.9 (v/v) and diluted 10 times. Samples (untreated curcumin control and PL-treated curcumin) and Trolox were dissolved in methanol and diluted to 10 and 20 μM, respectively. 0–40 μL of sample or Trolox was added to the wells of a 96-well black microplate, followed with addition of 75 mM PB to make up the volume to 100 μL per well. 60 μL of 200 nM FSS (prepared with 75 mM PB) was added to each sample well. Blank wells had 200 μL of 75 mM PB only and the negative control wells had 140 μL of 75 mM PB and 60 μL of 200 nM FSS, without antioxidant standard or sample. Triplicate wells were prepared for blank, assay negative control, and curcumin control and sample of different concentration levels. Microplates containing the above reagents were incubated in dark at 37˚C for 10 min. The peroxyl radical generator, AAPH, was prepared fresh with phosphate buffer to 60 mM, and added to the microplate after the 10-min incubation (40 μL per well, except for blank and negative control). All wells at this point had a final volume of 200 μL. Fluorescence intensity reading (Excitation = 485 nm, Emission = 527 nm) was obtained every min for 60 min using a TECAN Infinite M200 Pro plate reader (Männedorf, canton of Zürich, Switzerland). The area under curve (AUC) was calculated according to the following equation:

$$AUC=0.5+\sum_{i=2}^{59}\frac{A_{i}}{A_{1}}+0.5\times \frac{A_{60}}{A_{i}}$$ (4)

where A_1_ is the initial fluorescence reading at 1 min, A_60_ is the final fluorescence reading at 60 min, and A_*i*_ is the fluorescence reading at time *i* (*i* = 2–59 min).

The AUC was plotted against concentration of sample or Trolox (standard), which yielded a regression equation for each sample or Trolox. The slope of linear regression equation was used to determine the antioxidant capacity (ORAC values) of sample:

$$ORAC\;value=\frac{{Slope}_{Sample}}{{Slope}_{Trolox}}$$ (5)

ORAC values were expressed as μmol TE/μmol sample

### Cell culture

Caco-2 cells were cultured in tissue culture dishes (ØxH: 100 x 20 mm) with complete DMEM (DMEM supplemented with 10% FBS, 100 U/mL penicillin and 100 μg/mL streptomycin) in a 37˚C, 5% CO_2_, humidified incubator. Cells were maintained by changing media every 2–3 days and then sub-cultured at ~80% confluence, typically every 4–5 days [34]. Cells were seeded as per the required seeding density for the specific type of plates used in each assay, and cultured to differentiation, which took 21 days. Cells used in this study were in passages under 25.

### MTT assay

The 3-(4,5-dimethylthiazol-2-yl)-2,5-diphenyltetrazolium bromide (MTT) assay was performed to assess the cellular metabolic activity, as an indirect measure of cell viability of Caco-2 cells according to the procedures described by Liang et al. [32] Caco-2 cells were seeded in clear 96-well tissue culture-treated microplates at a density of 1×10^5^ cells/cm^2^ (i.e., 3.2×10^5^ cells/mL in 100 μL per 3.2-cm^2^ well), and cultured for 21 days to allow the formation of an epithelial monolayer (e.g., differentiation). Medium was changed every 2–3 days to maintain the cells. After 21 days, the cells were rinsed with PBS (pH 7.4) twice, prior to incubation with 100 μL sample (untreated or PL-treated curcumin at 50 μM in FBS-free DMEM; the concentration was determined from a concentration-dependent test with untreated curcumin in the concentration range of 0–500 μM) for 24 h. Negative control cells were incubated with FBS-free DMEM only. After treatment, the cells were washed with PBS twice, and incubated with MTT (0.5 mg/mL in FBS-free DMEM; 100 μL per well) for 4 h to allow the transformation of MTT to formazan by metabolically active cells. Then 100 μL of 10% SDS (0.1 M HCl) was added to dissolve the formazan crystals. The amount of formazan in the well was determined by measuring absorbance at 570 nm, using a Thermo Labsystems Multiskan Spectrum microplate spectrophotometer. Cell viability (% of control) was calculated from the equation:

$$MTT\;Relative\;Cell\;Viability\;(\%)=\frac{{Abs}_{570\;nm}(Sample)}{{Abs}_{570\;nm}\;(Negative\;Control)}\times 100$$ (6)

where Abs_570nm_ (Sample) is the absorbance of the cells treated with sample, Abs_570nm_ (Negative Control) is the absorbance of the negative control, which are cells incubated with media only.

### DCFH-DA assay for assessment of intracellular ROS inhibition

The capacity of control and PL-treated samples to mitigate induced peroxyl radical-initiated intracellular oxidative stress in Caco-2 cells was assessed using the dichloro-dihydro-fluorescein diacetate (DCFH-DA) assay described in Liang et al. [32] with modifications. Cells were seeded into 96-well black plates with a clear bottom, at a density of 3.2×10^5^ cells/mL with 100 μL per well. The cells were then cultured 21 days in DMEM supplemented with 10% FBS, 100 U/mL penicillin and 100 μg/mL streptomycin in a humidified incubator with 5% CO_2_ at 37˚C, with medium changes every 2–3 days. At day 21, differentiated cells were washed twice with PBS and exposed to 50 μM curcumin samples (untreated or PL-sample) in FBS-free DMEM for 24 h. This concentration was determined from a preliminary concentration-dependent test with 0–100 μM untreated curcumin standard. After 24h incubation, samples were removed and cells were rinsed with PBS twice, followed by addition of 5 μM DCFH-DA and incubation for 30 min at 37˚C in the dark, before addition of 1 mM AAPH. The cells were then continuously incubated for another hour, then fluorescence (excitation λ = 485 nm; emission λ = 527 nm) was measured with a Thermo Labsystems Multiskan Spectrum microplate spectrophotometer. Data was expressed as % ROS Inhibition using the following equation:

$$\%\;ROS\;Inhibition=\frac{Fluorescence(PC)-Fluorescence(Sample)}{Fluorescence(PC)-Fluorescence(NC)}\times 100$$ (7)

where Fluorescence(PC), Fluorescence(NC), and Fluorescence(Sample) refer to the fluorescence intensity from the cells that were treated with positive control (both DCFH-DA and AAPH), negative control (DCFH-DA only) and sample (prior to addition of DCFH-DA and AAPH) respectively.

### Glutathione assay for assessment of intracellular redox status

The preparation of cell samples for the glutathione assay followed the method described in Liang & Kitts [35] with modifications. Caco-2 cells were seeded in 6-well plates at a density of 4.75×10^5^ cells/mL with 2 mL each well and cultured for 21 days. At Day 21, cells were treated with samples (**dose-dependence test**: untreated curcumin at 0–100 μM in FBS-free DMEM; **PL effect test**: untreated and PL-treated curcumin at 50 μM in FBS-free DMEM) for 24 h. Then, cells were rinsed with PBS followed by addition of 2 mM H_2_O_2_ for 2 h to induce acute oxidative stress. Then, cells were rinsed three times with PBS. One mL of cold buffer (50 mM MES, pH 6–7, containing 1 mM EDTA) was added to each well, and cells were harvested using cell scrapers and collected into microcentrifuge tubes. Harvested cells were sonicated and freeze-thawed between -65˚C and room temperature for three times to facilitate lysis and homogenization. Total glutathione (GSH + GSSG) and oxidized glutathione (GSSG) values were measured using a GSH/GSSH Ratio Detection Assay Kit (Cayman Chemical, Ann Arbor, MI, USA) according to the manufacturer’s protocol. The amount of reduced GSH was calculated by subtracting the amount of GSSG from that of total glutathione. The ratio of reduced to oxidized glutathione (GSH/GSSG) will be calculated as an indicator of intracellular redox status.

### Statistical analysis

Biological triplicates (*n* = 3) of samples were prepared by performing PL treatment three times, using the fluence levels presented in **Table 1,** but in random order each time. Technical triplicate measurements were performed for all analyses and assays, respectively. All data was expressed as mean ± SD. One-way ANOVA (α = 0.05) was performed to determine if a significant difference existed among treatment groups. Post hoc Tukey’s HSD test was performed to identify statistically significantly different groups. In the case where no significant difference was identified using one-way ANOVA, pairwise two-tailed *t*-tests (α = 0.05) were done to compare individual PL-treated curcumin samples with the untreated control. Pearson’s correlation analysis was performed between the curcumin dimer content (peak area ratio [curcumin dimer/d_6_-curcumin]) and pulsed light fluence (J/cm^2^), chemical- and cell-based antioxidant capacity results.

## Results

### PL induced reduction in curcumin content in a fluence-dependent manner

**Fig 1:**
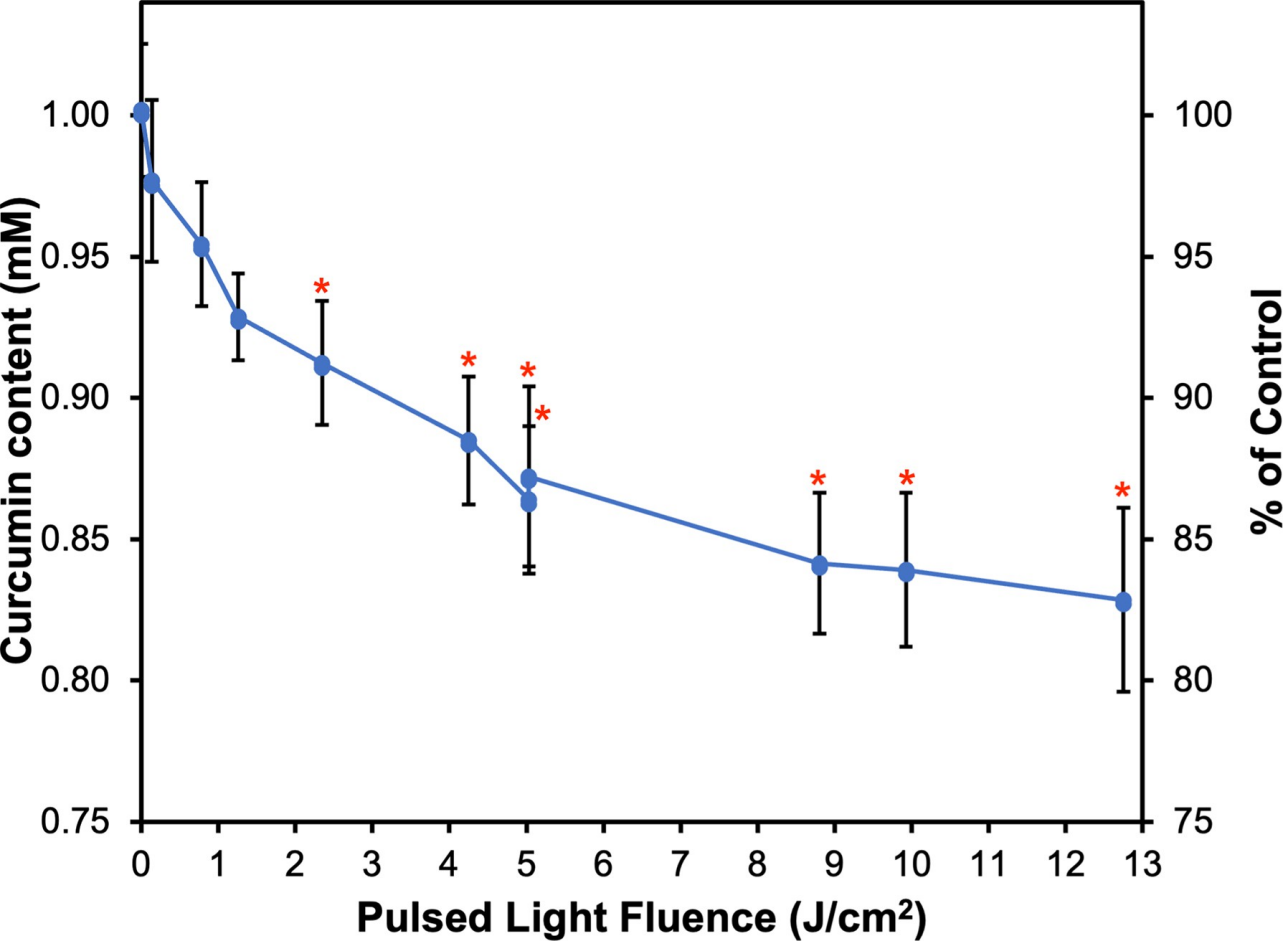
Curcumin content in pulsed light-treated curcumin samples and untreated control. Data is presented as mean ± SD (n = 3). A red asterisk indicates significant difference (*P* < 0.0001) from untreated control (0 J/cm^2^).

The curcumin concentration, determined by HPLC-DAD, in untreated control and samples treated with PL at proposed fluence levels are presented in **Fig 1**. All samples treated by PL exhibited lower (*P* < 0.05) curcumin content compared to the untreated control (1.002 ± 0.024 mM curcumin), and fluence level was positively correlated with the extent of reduction (Pearson correlation coefficient *r* = 0.91, *P* < 0.0001). Statistically significant reductions of curcumin were observed at fluence ≥ 2.35 J/cm^2^, with ≤ 0.912 mM curcumin remaining (91% of control). At the highest fluence level (12.75 J/cm^2^), 0.829 ± 0.033 mM curcumin was retained, which indicated a 17% reduction in curcumin content. Temperature change was positively correlated with fluence level (*r* = 0.96, *P* < 0.0001), but the increment of change was within 5˚C, so it was assumed that no thermal degradation of curcumin was induced by the PL treatment in this study.

### Identification of curcumin photo-transformation product

Using the HPLC/QTOF conditions described in **Identification of potential curcumin photo-transformation products**, the identification of curcumin was confirmed at a retention time of around 18.895 min (**S1 Fig** in S1 File). With the most abundant ion m/z in the range of 369.1343–369.1353 for control and PL-treated samples (mass accuracy of 1.35–4.06 ppm, respectively) (**S1B and S1C Fig** in S1 File), and with a mass error < 5 ppm, the identification was deemed accurate. When this ion was further analyzed in MS/MS mode (**S2A Fig** in S1 File), the major product ions were 145.0267, 177.0533, 117.0323 (**S2B Fig** in S1 File), which corresponded to the fragmentation pattern of curcumin reported in the literature;[36] these ions have already indicated the presence of ferulic acid structure in fragmentation patterns [31,37]. Semi-quantification, as peak area to internal standard ratio, of curcumin using 10 μg/mL d_6_-curcumin as an internal standard provided a double confirmation (**Table 2**) for the HPLC-DAD quantification results presented in **Fig 1**.

**Table 2:** Peak areas of curcumin, curcumin dimer (tentative), and d_6_-curcumin in untreated and PL-treated curcumin samples determined by HPLC/QTOF in MS mode.

		Curcumin	Curcumin Dimer	d_6_-Curcumin	Ratio (Curcumin/ d_6_-Curcumin)	Ratio (Curcumin Dimer/ d_6_-Curcumin)
**Target m/z**		369.1333	735.2436	372.1685		
**Ret. time (min)**		18.895	18.530	18.846		
**Fluence (J/cm** ^**2**^ **)**	**0**	53,810,584	ND	14,579,425	3.69	N/A
	**0.14**	49,788,725	ND	13,905,527	3.58	N/A
	**0.78**	49,931,107	20,923	14,024,956	3.56	0.0014
	**1.26**	50,568,705	57,477	14,309,538	3.53	0.0040
	**2.35**	48,377,994	78,312	13,972,453	3.46	0.0056
	**4.25**	48,298,526	97,508	14,205,257	3.40	0.0068
	**5.03**	48,112,656	117,860	14,333,730	3.36	0.0082
	**5.03**	45,902,719	114,028	13,633,358	3.37	0.0083
	**8.81**	43,694,038	232,782	14,162,239	3.28	0.016
	**9.93**	47,101,588	250,865	14,347,734	3.09	0.017
	**12.75**	40,789,529	301,131	14,467,669	2.82	0.021

ND = Not detected; N/A = Not applicable.

**Fig 2:**
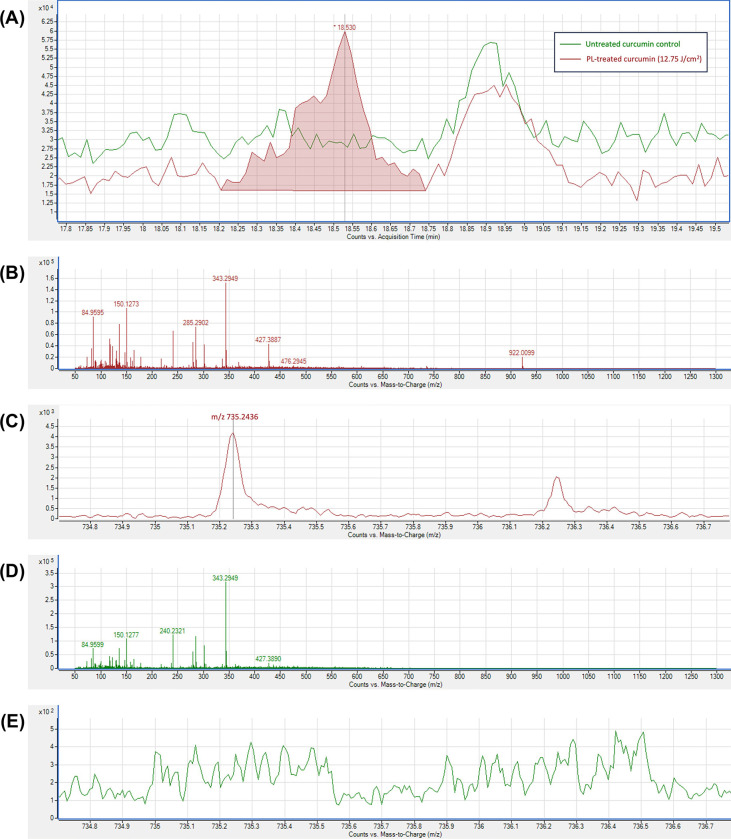
HPLC/QTOF-MS Analysis of untreated curcumin control (green) and PL-treated curcumin at a fluence of 12.75 J/cm^2^ (red). (A) HPLC/QTOF Chromatogram (+ESI EIC 735.2436) of untreated curcumin control (green line) and PL-treated curcumin at a fluence of 12.75 J/cm^2^ (red line). (B) Mass spectrum detected in MS mode of the peak at 18.530 min of PL-treated curcumin with (C) close-up to show the m/z 735.2436. (D) Mass spectrum in MS mode of untreated control at 18.530 min showed no peak of m/z 735.2436 (E).

**Fig 3:**
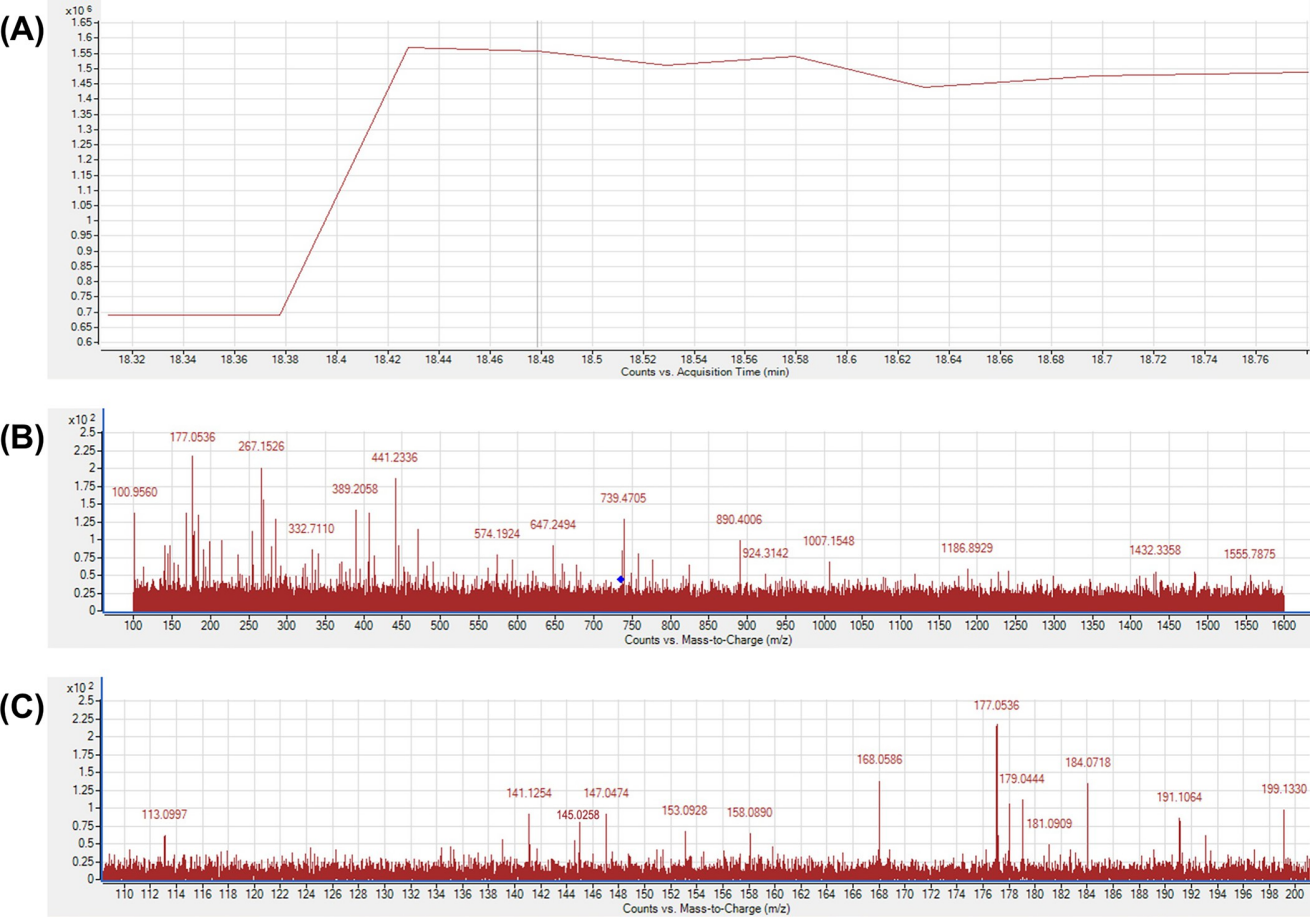
HPLC/QTOF-MS/MS Analysis of PL-treated curcumin at a fluence of 12.75 J/cm^2^. (A) HPLC/QTOF chromatogram (+ESI TIC). (B) Mass spectrum detected in MS/MS mode of the area of elution of the potential curcumin dimer (m/z 735.2436; retention time 18.530 min), with (C) close-up to show the m/z range of 110–200.

**Fig 4:**
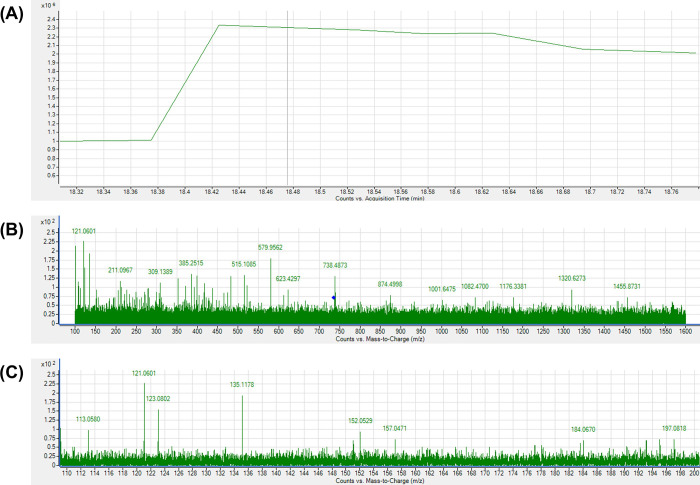
HPLC/QTOF-MS/MS Analysis of untreated curcumin control. (A) HPLC/QTOF chromatogram (+ESI TIC). (B) Mass spectrum detected in MS/MS mode of the area of elution of the potential curcumin dimer (m/z 735.2436; retention time 18.530 min), with (C) close-up to show the m/z range of 110–200.

Expected photo-transformation products, for which analytical standards were available (**S1 Table** in S1 File), including vanillin, vanillic acid, ferulic acid, ferulic aldehyde, and feruloyl methane, were not detected in either untreated curcumin control or PL-treated curcumin samples. However, potential photo-transformation products (**S3 Table** in S1 File) for which analytical standards were not available were screened on chromatograms with theoretical m/z values. Only the chromatogram of m/z 735.2436 showed a noticeable difference between untreated control and PL-treated samples (**Fig 2A).** This observation was noted with the highest fluence level PL-treated sample. The ion m/z 735.2436 peak was detected at 18.530 min in all PL-treated samples, except for the lowest fluence group (0.14 J/cm^2^), but not detected in the untreated control. The mass spectrum at 18.530 min also confirmed the presence ion 735.2436 in most PL-treated samples (**Fig 2B and 2C**) and the absence in the control (**Fig 2D and 2E**). Therefore, this ion was selected for further MS/MS analysis (**Fig 3A**) which revealed a defined peak (~18.479 min). The presence of ions 177.0536 and 145.0258 in the MS/MS spectrum (**Fig 3B and 3C**) confirmed that the parent ion had a ferulic acid structure similar to curcumin, and because the m/z value of the parent ion matched with the theoretical value (m/z 735.2436) with a mass error < 3 ppm, it was speculated that this was a curcumin dimer. The chromatogram and MS/MS spectra of untreated curcumin control (**Fig 4**) confirmed that curcumin dimers were not present in the untreated sample. By integrating the peaks at ~18.5 min (**Fig 2A** as an example), the content of curcumin dimer was semi-quantified, as peak area to internal standard ratio, against internal standard (d_6_-curcumin, 10 μg/mL) and is shown in **Table 2**. Dimerization of curcumin, attributed to PL treatment above the very lowest fluence treatment was confirmed with the content of curcumin dimer being positively correlated with PL fluence (*r* = 0.992, *P* < 0.0001).

### PL treatment altered chemical-based antioxidant capacity of curcumin

**Fig 5:**
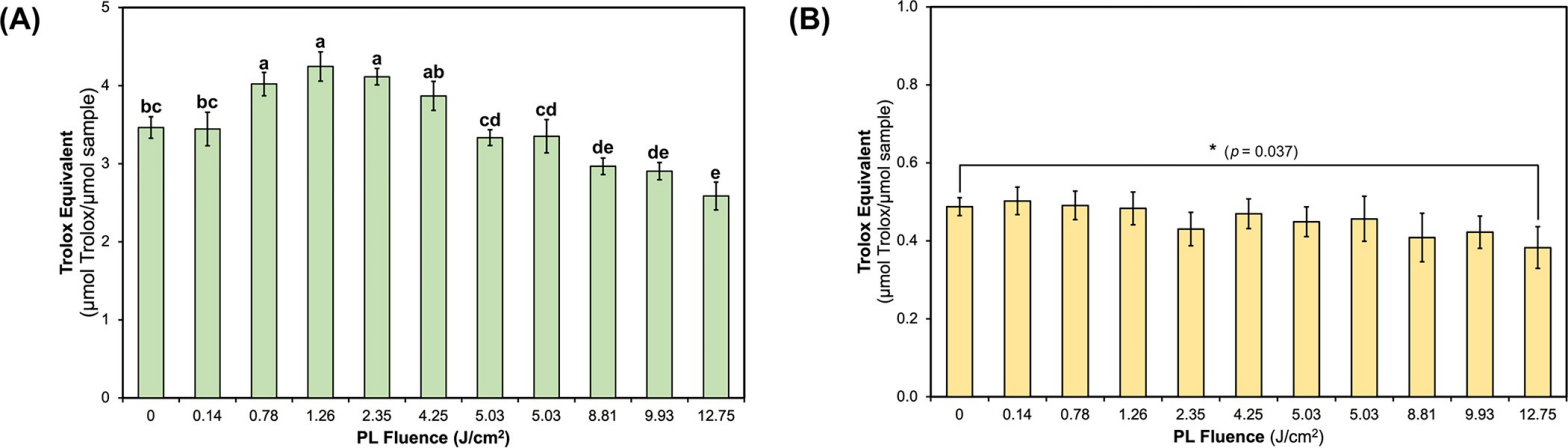
Chemical-based antioxidant capacity of untreated and PL-treated curcumin, determined by ABTS assay (A) and ORAC assay (B). Data is presented as mean ± SD (n = 3). In (A), letters a-e denote significantly different (*P* < 0.0001) groups by one-way ANOVA; in (B), no significant difference was identified by one-way ANOVA, but an asterisk (*) denotes the treatment group that was significantly different (*P* < 0.05) from control by *t*-test.

All PL-treated samples, except for the sample treated with the lowest fluence 0.14 J/cm^2^, altered antioxidant capacity compared to the untreated control, using the ABTS^•+^ scavenging assay (**Fig 5A**). The relatively low fluence levels (e.g., 0.78–2.35 J/cm^2^) resulted in significantly higher (*P* < 0.0001) ABTS^•+^ scavenging capacity of curcumin, while the relatively high fluence levels (e.g., 8.81–12.75 J/cm^2^) resulted in significantly lower (*P* < 0.0001) capacity. The sample group treated with the highest fluence (12.75 J/cm^2^) had the lowest relative ABTS^•+^ scavenging capacity. The subtle transformation of curcumin to generate a curcumin dimer was negatively correlated (*r* = -0.76; *P* = 0.006) with the chemical Trolox ABTS^•+^ scavenging capacity.

In the ORAC assay (**Fig 5B**), statistical significance was only marginal among control and PL-treated groups using one-way ANOVA (*P* = 0.053). Re-analysis of ORAC results from individual treatment groups compared against the control using a *t-*test, showed that the group treated with the highest fluence (12.75 J/cm^2^) produced a significant lower (*P* = 0.036) antioxidant capacity to protect against peroxyl radical formation. A Pearson’s correlation analysis of this outcome confirmed that the PL generation of a curcumin dimer adversely affected ORAC antioxidant capacity (*r* = -0.94; *P* < 0.001).

### Effect of PL treatment of curcumin on MTT activity in differentiated Caco-2 cells

**Fig 6:**
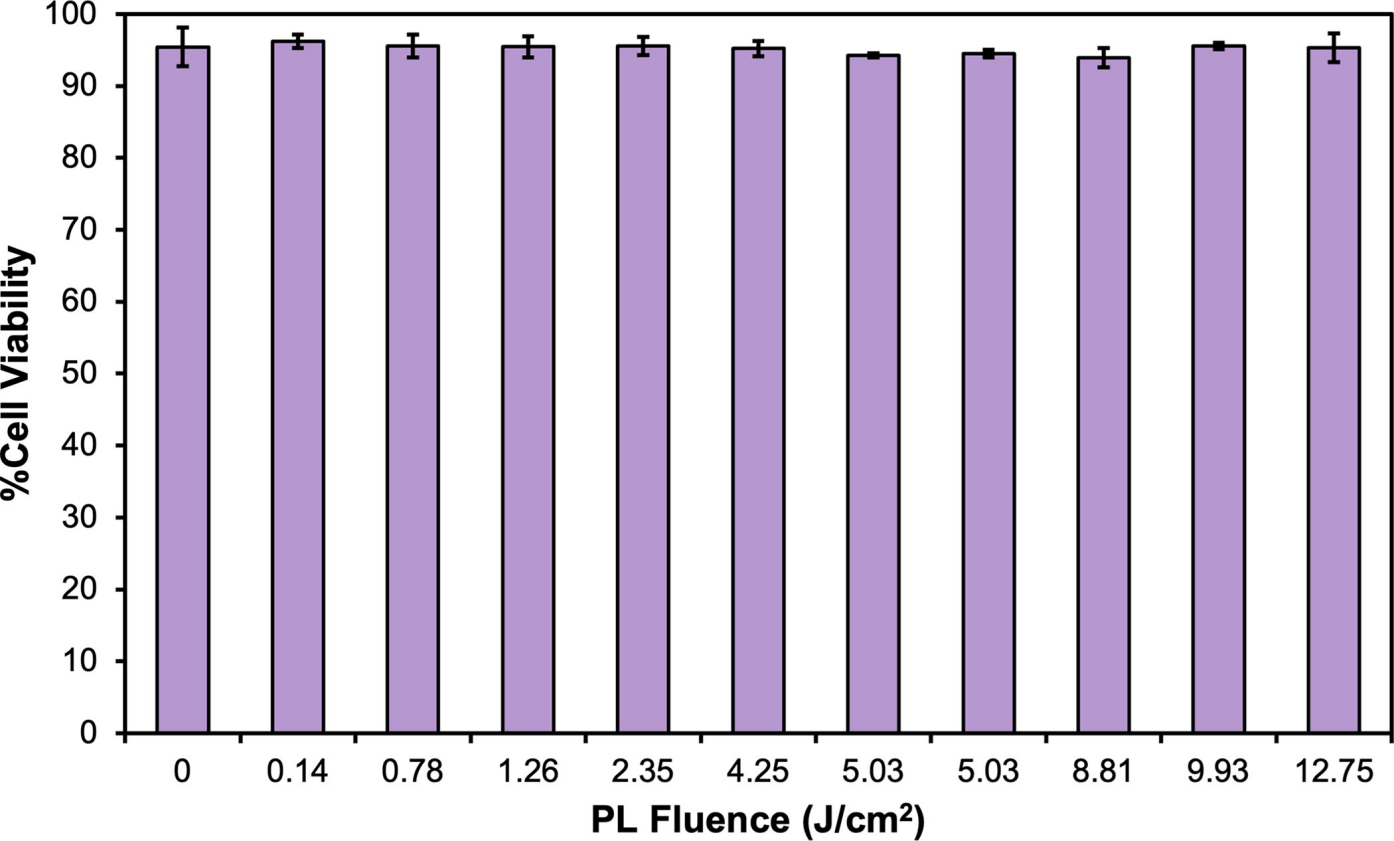
Cell viability (%Control) of differentiated Caco-2 cells treated by curcumin control and PL-treated curcumin. Data is presented as mean ± SD (*n* = 3). No statistical significance was found among groups (*P* = 0.73).

At 50 μM, untreated curcumin control (0 J/cm^2^) and PL-treated curcumin samples at all fluence levels (0.14–12.75 J/cm^2^) produced comparable cell viability, assessed using the MTT assay (e.g. higher than 93%) (**Fig 6**). It was concluded that curcumin treated by PL within 12.75 J/cm^2^ did not result in significant cytotoxicity of differentiated Caco-2 cells.

### Effect of PL treatment on antioxidant capacity of curcumin in cultured, differentiated Caco-2 cells

**Fig 7:**
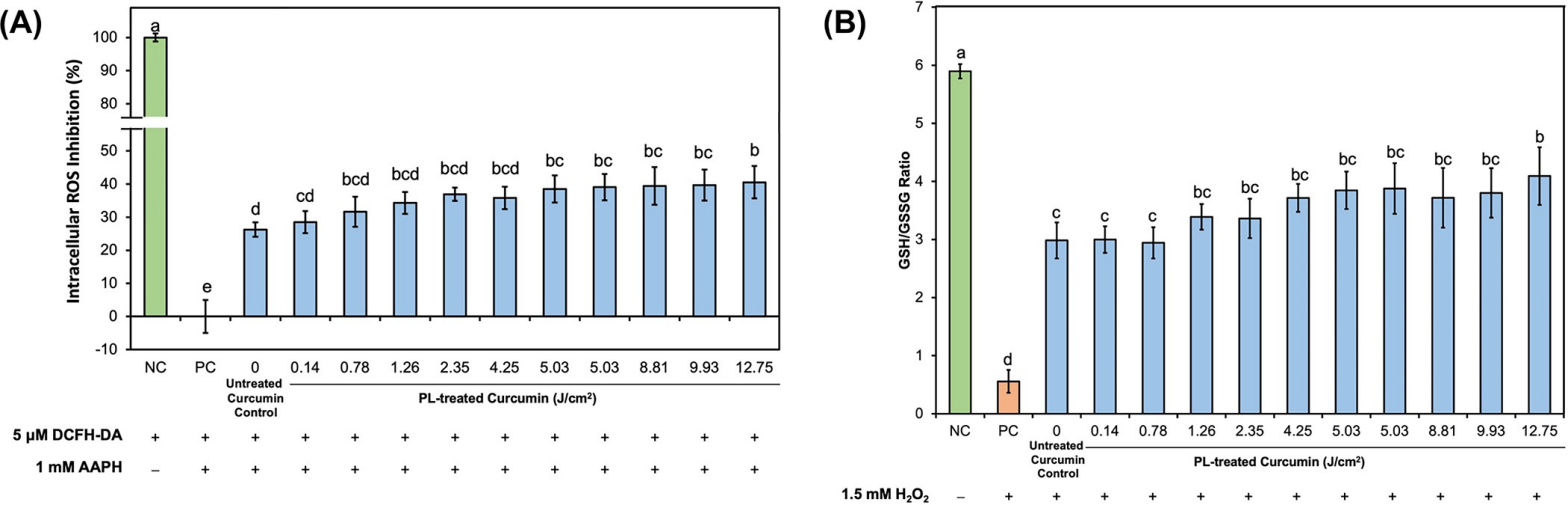
Intracellular antioxidant capacity of 50 μM untreated and PL-treated curcumin in Caco-2 cells, determined by (A) DCFH-DA assay and (B) glutathione assay. Data is presented as mean ± SD (*n* = 3). Letters a-d denote significantly different (*P* < 0.0001) groups. NC = negative control, referring to the cells not treated with antioxidant pre-treatment nor oxidative stress inducer (AAPH in DCFH-DA assay or H_2_O_2_ in glutathione assay); PC = positive control, referring to the cells with oxidative stress inducer but without antioxidant pre-treatment. “0 J/cm^2^” refers to cells treated with untreated curcumin control.

**Fig 7A** presents the cell-based antioxidant capacity assessment of untreated and PL-treated curcumin samples used in differentiated Caco-2 cells to quantify the intracellular ROS inhibition after the application of a peroxyl radical generator AAPH (DCFH-DA assay) inducer. The corresponding change in intracellular GSH/GSSG ratio, used as an indicator of oxidative stress induced by 1.5 mM H_2_O_2_ was significantly reduced by curcumin (**Fig 7B**) without significantly affecting cell viability (98% of control). For example, whereas cells not treated by curcumin showed a decrease in AAPH-induced intracellular ROS production by 26.24 ± 2.15%, the corresponding H_2_O_2_-change in GSH/GSSG ratio was restored to about 50% of the original level, thus confirming the antioxidant capacity of curcumin in differentiated Caco-2 cells. PL-treated curcumin samples had a comparable or higher antioxidant effect, which was positively correlated with fluence level (DCFH-DA assay: *r* = 0.828, *P* = 0.002; glutathione assay: *r* = 0.859, *P* = 0.0007). In the DCFH-DA assay, curcumin treated with PL at fluence levels ≥ 5.03 J/cm^2^ produced a significantly higher (*P* < 0.0001) inhibitory effect against intracellular ROS generation. In the glutathione assay, the increase in antioxidant capacity was not significant until a fluence of 12. 75 J/cm^2^ was reached. In both assays, curcumin treated with the highest fluence (12.75 J/cm^2^) resulted in the strongest antioxidant effect. Results from both assays strongly agreed with each other (*r* = 0.927, *P* < 0.0001). A Pearson correlation analysis confirmed that both DCFH-DA and GSH/GSSG outcomes in cultured Caco-2 cells were identically positively correlated with the appearance of the curcumin dimer following PL treatment of curcumin (*r* = 0.85; *P* = 0.009).

## Discussion

Quantification of changes in curcumin content resulting from photosensitivity induced by PL treatment was useful to confirm losses of curcumin that could be attributed to chemical transformation and provide an explanation for the observed changes in antioxidant potential in both chemical and biological assays, respectively. The fluence, a parameter that describes the total energy dose applied to the sample, was an important factor for assessing the degree of chemical transformation of curcumin. PL within the usual application range of fluence did not initiate the breakdown of curcumin into smaller phenolic molecules, such as vanillin, vanillic acid, ferulic acid, ferulic aldehyde, and feruloylmethane, that are commonly reported as the main products of photochemical hydrolytic degradation of curcumin [26]. The products from the major oxidative decomposition pathway of curcumin, such as bicyclopentadione [7,8], were also not identified, but rather the use of PL in this study showed the induced dimerization of curcumin.

Based on current literature, dimerization is a minor transformation pathway of curcumin upon oxidation, which involves the formation of curcumin phenoxyl radical via autooxidation or attack by ROS, relocation of the radical within the curcumin structure, and coupling of two curcumin radicals to neutralize the unstable structures. The first step can be triggered by oxidating agents, or photoactivation [7,25]. In this study, we report that the PL treatment provided the activation energy required to initiate the formation of curcumin radicals, which in turn formed dimers that are more thermodynamically stable. The number of phenolic (–OH) groups in the curcumin dimers thus formed depend on the location of the radical on both monomers [29,30]. For example, if the radical resides in the benzene ring and phenolic structure converts to a quinone methide structure, one phenolic–OH group is lost in the final structure. Most of the identified curcumin dimers, though, have four phenolic groups retained in the final structure [29,30]. The ortho-methoxy groups remained intact in the dimerization process.

A previous study revealed the formation mechanism of curcumin dimers and furthermore the contribution of this process to the antioxidant activity of curcumin against lipid peroxidation [30]. After scavenging a lipid hydroperoxyl radical and breaking the lipid peroxidation chain reactions, each curcumin molecule bears a radical which can react with another curcumin radical via a radical-radical coupling reaction, and the coupling products tend to undergo internal cyclization to form stable radical termination products. Another study reported the formation of curcumin dimers in the radical-scavenging activity of curcumin against DPPH free radicals [29].

In this study, the results of both chemical-based antioxidant assays suggested diminished antioxidant capacity after curcumin dimerization. The ORAC assay is based on the hydrogen atom transfer (HAT) as the antioxidant mechanism [38]. The phenolic groups in curcumin structure likely contribute to the HAT that can be further enhanced by the ortho-methoxy groups [39]. The ABTS^•+^ scavenging assay, on the other hand, is based on the single electron transfer (SET) from antioxidant compounds to neutralize the pre-generated N-centered radical cation [40]. The reductions in radical scavenging capacity with the increase in the content of curcumin dimers resulting from higher intensity of PL treatment may be related to the changes in stereochemistry. Curcumin dimers are bulkier in molecular size than the monomer, and thus steric hindrance (especially when the dimer molecules are in close proximity with the free radical) limits the reactivity for radical scavenging.

Of importance was the finding that curcumin dimerization did not induce cytotoxicity in differentiated Caco-2 cells; however, a quality to potentiate a greater antioxidant capacity against AAPH-induced intracellular ROS production and with H_2_O_2_-induced oxidative stress was observed. This is a novel finding, as there are no published studies on the cellular antioxidant activity of curcumin dimers, or the role of dimerization in the cellular antioxidant activity of curcumin. This study provides insight into the *in vitro* antioxidant potential of curcumin dimers in a cell-based model of human intestinal epithelium.

Studies have shown that upon entering the intracellular space of non-tumor cells via passive diffusion [41], curcumin regulates the cellular redox status and mitigates oxidative stress by scavenging intracellular free radicals [42]. As mentioned, one curcumin dimer molecule was formed from two curcumin monomers producing a diverse structure of existing phenolic groups that yielded subtle changes in cellular redox bioactivity. The apparent enhancement of intracellular antioxidant capacity observed with curcumin dimerized products using Caco-2 cells in the present study agrees with the reports of others that curcumin targets the Keap-1-Nrf2 pathway to promote activation and expression of antioxidant genes and upregulation of key antioxidant enzymes [43,44]. Hence, while it is generally believed that the phenolic groups present in the curcumin structure are responsible for scavenging the intracellular free radicals, the activity of altered functional group(s) in curcumin on upregulating activity of specific antioxidant enzymes or antioxidant signaling pathways have not yet been demonstrated. Future studies are warranted to assess the Caco-2 cell permeability and related intracellular cell signaling of curcumin dimers to define the mechanism for antioxidant capacity.

## Conclusion

Within the typical application range of fluence, pulsed light decreased curcumin content in a pure methanolic curcumin solution in a fluence-dependent manner. However, the reduction in curcumin content did not directly correlate with a magnitude of change expected in antioxidant capacity. In chemical antioxidant ABTS^•+^ (SET) and ORAC (HAT) radical scavenging assays, higher PL fluence was correlated with a significantly lower antioxidant capacity; whereas in cell-based assays, PL fluence had a more defined, positive correlation with the intracellular antioxidant capacity of curcumin. HPLC-QTOF-MS and HPLC-QTOF-MS/MS analyses revealed that curcumin dimers were the only detectable transformation products, indicating a higher potential biological antioxidant potency attributed to the dimers. In lieu of the fact that bioavailability of curcumin will be dependent on both the bioaccessiblity character and bioactivity potential, future studies are needed to include the complexity of a food matrix to shown possible antagonistic or synergistic interaction with curcumin dimers.

## Supporting information

S1 File Supporting information. (DOCX)
