# Supplementary material for: Effect of pulsed light on curcumin chemical stability and antioxidant capacity
Source: PLoS One. 2023 Sep 1;18(9):e0291000. doi: 10.1371/journal.pone.0291000 (PMC10473471; doi:10.1371/journal.pone.0291000)
Supplement: S1 File — (DOCX) [file pone.0291000.s001.docx]

***Supplementary Information***

**Effect of Pulsed Light on Curcumin Chemical Stability and Antioxidant Capacity**

Huiying Amelie Zhang and David D. Kitts*

Food, Nutrition and Health, Faculty of Land of Food Systems, University of British Columbia, Vancouver, BC V6T 1Z4, Canada

Table S1. Chromatographic (retention times) and mass spectrometric quantification parameters (target ion m/z) of reference compounds in HPLC/QTOF-MS/MS Analysis

| Compound | Retention Time (min) | Target Ion m/z | Target Ion Structure |
| --- | --- | --- | --- |
| Curcumin | 18.895 | 369.1333 | [M+H]^+^ |
| d_6_-Curcumin | 18.846 | 375.1685 | [M+H]^+^ |
| Vanillin | 7.181 | 153.0546 | [M+H]^+^ |
| d_3_-Vanillin | 7.439 | 156.0719 | [M+H]^+^ |
| Vanillic acid | 5.165 | 151.0839 | [M-H_2_O+H]^+^ |
| Ferulic acid | 8.482 | 177.0547 | [M-H_2_O+H]^+^ |
| Ferulic aldehyde | 10.059 | 177.0557 | [M+H]^+^ |
| Feruloyl methane | 11.002 | 191.0714 | [M+H]^+^ |

Reference: Tønnesen et al. (1986).

Table S2. Solvent gradient in HPLC/QTOF analysis for curcumin and relevant compounds

| Time [min] | Solvent A [%] | Solvent B [%] |
| --- | --- | --- |
| 0 | 100 | 0 |
| 1 | 94 | 6 |
| 4 | 91 | 9 |
| 6 | 87 | 13 |
| 14 | 65 | 35 |
| 25 | 10 | 90 |
| 30 | 10 | 90 |
| 32 | 100 | 0 |

Table S3. Compounds as potential photo-transformed products of curcumin, for which analytical standards were not available

| **Compound** | **Theoretical m/z** | **Ion Structure** |
| --- | --- | --- |
| 4-Vinylguaiacol | 151.0759 | [M+H]^+^ |
| Diguaiacol | 247.0965 | [M+H]^+^ |
| Cyclobutylcyclopentadione | 369.1333 | [M+H]^+^ |
| Bicyclopetadione | 401.1231 | [M+H]^+^ |
| Ketohydroxycyclopentadione | 401.1231 | [M+H]^+^ |
| Hemiacetalcyclopentadione | 401.1231 | [M+H]^+^ |
| Dihydrocyclopentadione | 403.1387 | [M+H]^+^ |
| Curcumin dimers | 735.2436 | [M+H]^+^ |

References: Fujisawa et al. (2004); Gordon et al. (2015); Schneider et al. (2015); Tønnesen et al. (1986).

| **(A)** | 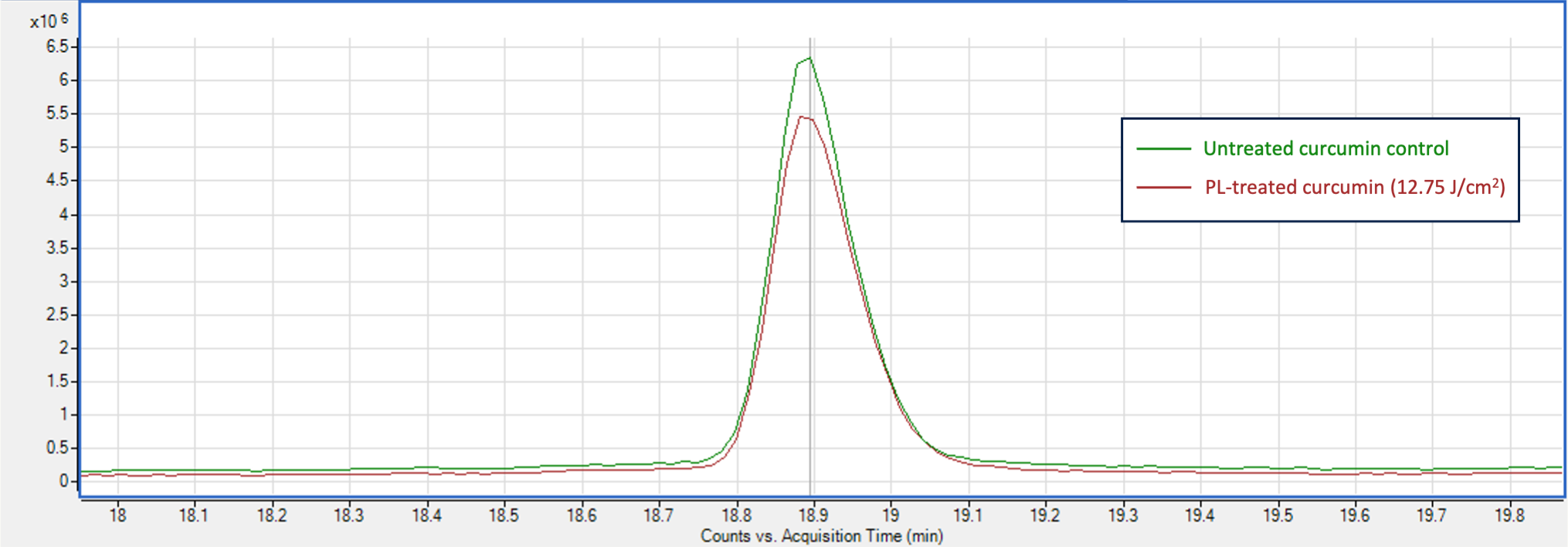 |
| --- | --- |
| **(B)** | 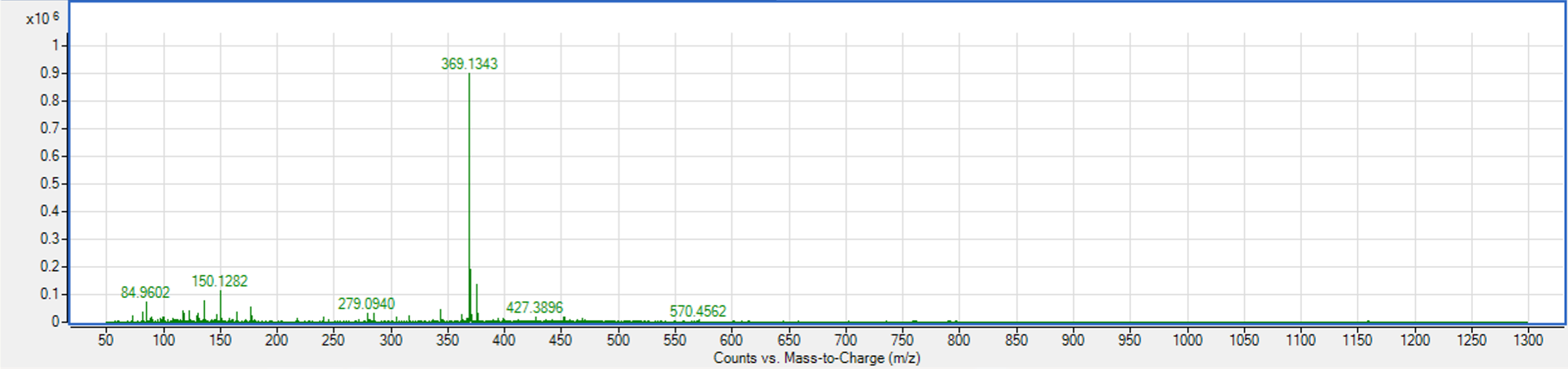 |
| **(C)** | 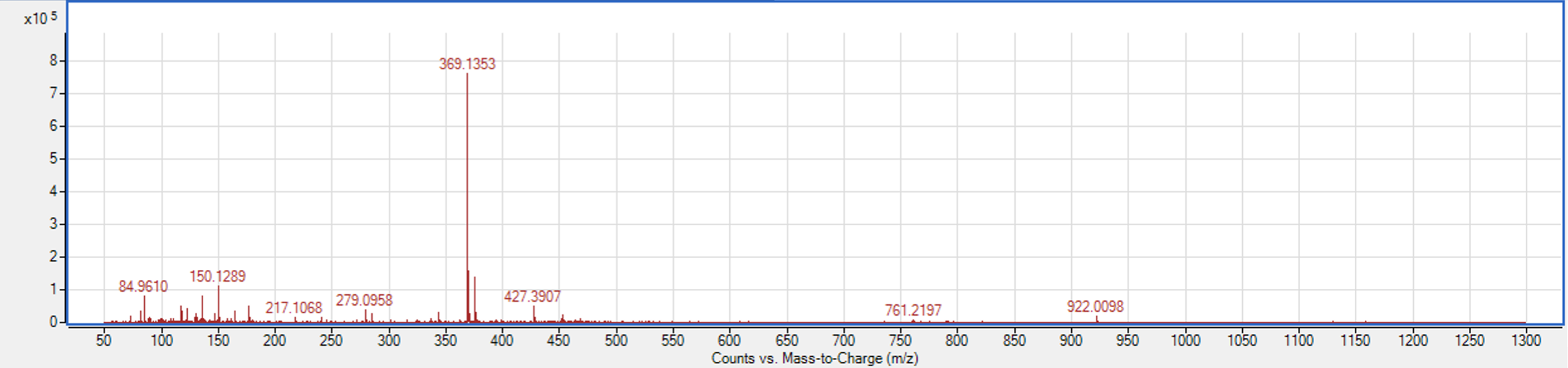 |

**Figure S1. (A) HPLC/QTOF chromatogram (+ESI EIC 369.1333) of untreated curcumin control (green line) and PL-treated curcumin at a fluence of 12.75 J/cm^2^ (red line); and mass spectra detected in MS mode of the peak at 18.895 min of untreated control (B) and PL-treated curcumin (C)**

| **(A)** | 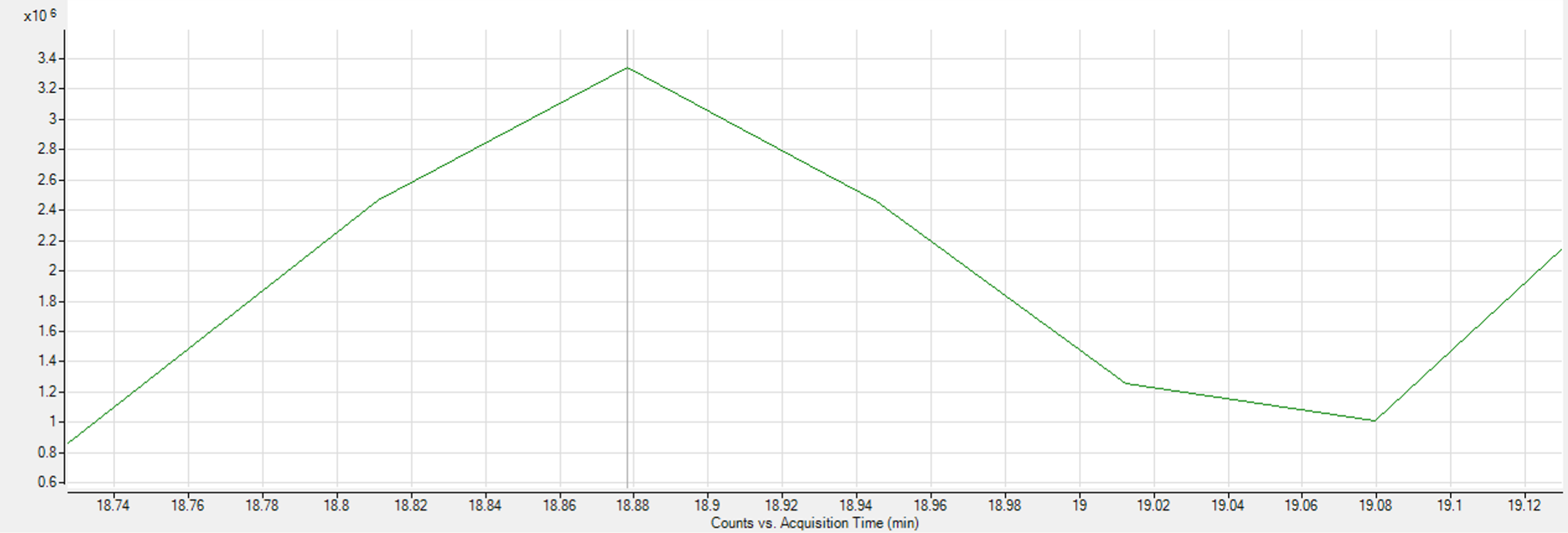 |
| --- | --- |
| **(B)** | 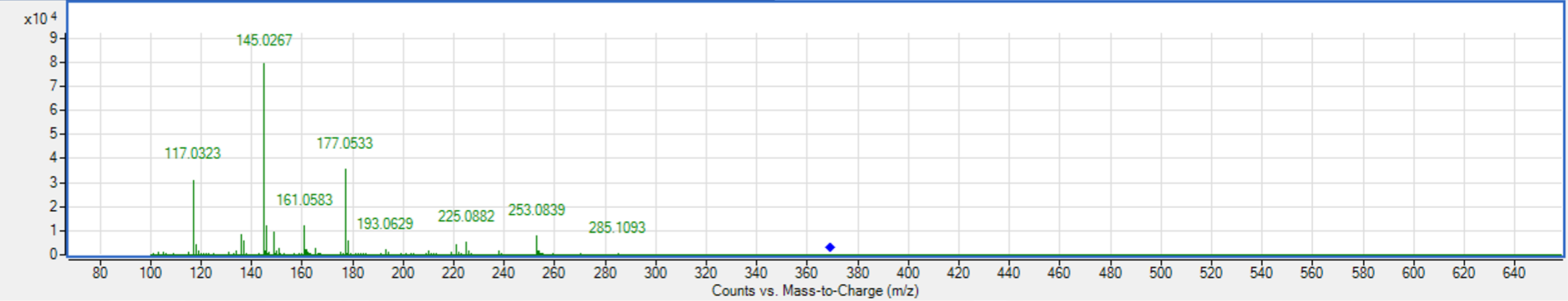 |

**Figure S2. HPLC/QTOF chromatogram (+ESI TIC) (A) and mass spectrum (B) of untreated curcumin control detected in MS/MS mode (parent ion m/z 369.1343).**
